# Current Status of Tricuspid Valve Interventions in Asia Pacific Region

**DOI:** 10.1016/j.jacasi.2024.10.008

**Published:** 2024-12-17

**Authors:** Kent Chak-yu So, Jianqiang Xu, Kevin Ka-ho Kam, Shih-Hsien Sung, Krissada Meemook, Dee Dee Wang, Gilbert H.L. Tang, Alex Pui-wai Lee, Yat-yin Lam

**Affiliations:** a Department of Medicine and Therapeutics, Prince of Wales Hospital, Chinese University of Hong Kong, Hong Kong SAR, China; b Department of Cardiology, Tianjin First Central Hospital, Tianjin, China; c Institute of Emergency and Critical Care Medicine, National Yang Ming Chiao Tung University, Taipei, Taiwan; d Department of Medicine, Faculty of Medicine at Ramathibodi Hospital, Mahidol University, Bangkok, Thailand; e Wayne State University School of Medicine, Detroit, Michigan, USA; f Department of Cardiovascular Surgery, Mount Sinai Health System, New York, New York, USA; g Laboratory of Cardiac Imaging and 3D Printing, Li Ka Shing Institute of Health Sciences, Shatin, New Territtories, Hong Kong SAR, China; h Central Medical, Hong Kong SAR, China

**Keywords:** Asia Pacific, transcatheter annuloplasty, transcatheter edge-to-edge repair, transcatheter tricuspid valve replacement, tricuspid regurgitation

## Abstract

Transcatheter tricuspid interventions are becoming increasingly more common in Asia Pacific. In the past decade, clinicians in Asia Pacific have worked with a multitude of new transcatheter tricuspid technologies. A standardized clinical algorithm to diagnose symptomatic tricuspid regurgitation to increase patient access to novel right heart therapies has not yet been identified. Anatomic diversity in the Asia Pacific patient population; disease prevalence patterns; and socioeconomic, cultural, and local health structures represent unique challenges in the treatment of these patients with right heart failure. As advancements are made in right heart failure and transcatheter tricuspid technologies, hopefully more patients can be treated not just in Asia Pacific, but across the entire world.

## Highlights

• In the past decade, many novel transcatheter tricuspid right heart failure therapies have been developed.
• There are unique challenges to scaling the treatment of tricuspid valve disease in Asia Pacific, requiring increased patient awareness and diagnosis, improving health care economics, and investment in physician training.
• Standardized algorithms for treating TR will increase patient access to potentially lifesaving therapies.

Symptomatic severe tricuspid regurgitation (TR) has significant clinical and economic impact at the patient and societal level.^1^ In the Asia Pacific region (APAC), open heart surgery for isolated TR is not commonly performed because of the high operative risk (8%-10% mortality).^2^, ^3^, ^4^ There is a need for transcatheter interventions for TR for patients not eligible for open-heart surgery. As we have learned from the mitral space, the mechanisms of treatment are complex and even more so in the tricuspid valve. The right heart has a multichamber anatomy that is nontubular in shape. Therefore, the transcatheter technologies require dedicated anchoring mechanisms, paravalvular shield guards, and potentially novel mechanisms for delivery to the landing zone.^5^ Moreover, in recent years, a wide range of novel technologies have been developed.^6^ In this review, we aim to summarize the latest developments in transcatheter tricuspid valve (TV) interventions with a specific focus on patients in the APAC region.

## Disease Burden

In APAC, the prevalence of TR is similar to North American and European reported registries (Central Illustration). In the United States, the prevalence of moderate or severe TR is estimated at 0.55%, with higher prevalence in women and the elderly.^7^ In a Chinese national survey of 31,499 people, the prevalence of TR was 0.8%.^8^ The China DVD (China ElDerly Valve Disease) study, evaluating 8,929 hospitalized patients over the age of 60 years, found isolated TR to be the second most common native valvular heart disease (16.5% TR, following mitral regurgitation at 26.9%).^9^ In South Korea’s nationwide hospital-based registry of 4,089 patients, tricuspid valve disease was the third most common valvular heart disease (28.6% of patients).^10^ A retrospective analysis of the Japanese population using administrative claims data from acute care hospitals in 2019 found among 203,398 patients with valvular heart disease, tricuspid valve disease ranked third (22.9%) in overall prevalence (mitral valve disease was 49.0%, aortic valve disease was 44.9%, pulmonic valve was 2.2%, and 14.9% of patients had more than 1 disordered valve).^11^ Gossl et al^12^ demonstrated in a community-based prospective study 16% of patients ≥65 years of age had previously undiagnosed moderate-severe valvular heart disease; of which TR made up 7.2% of this patient population. Hence, the true prevalence of tricuspid regurgitation across communities is unclear.

**Central Illustration:**
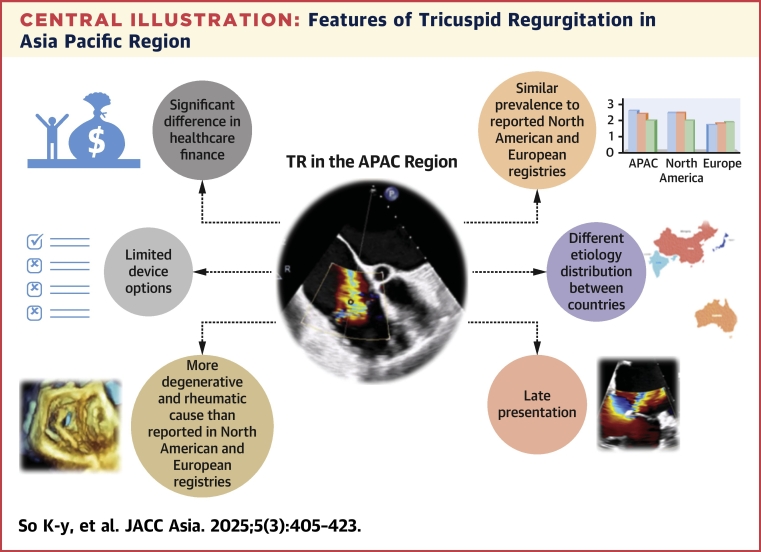
Features of Tricuspid Regurgitation in Asia Pacific Region Although the prevalence of tricuspid regurgitation (TR) across the Asia Pacific region (APAC) nations is similar to the prevalence reported in European and North (N) American registries, there is a greater prevalence of degenerative and rheumatic TR among the APAC nations. The diversity of health care infrastructure and socioeconomic challenges amongst the different APAC countries affect patient access to new technologies and treatment options.

TR is associated with increased morbidity and mortality.^1^ Data from the UK Biobank indicates that patients with TR have 2.5 times higher risk of all-cause mortality compared with those without TR.^13^ Outcome data of TR in APAC is limited. A study by Yang et al^14^ found that Chinese patients with multivalvular heart disease involving the left and right heart have different survivals depending on the degree of surgical correction performed at time of their initial valvular operation. Patients receiving intervention to correct significant TR at the time of open-heart surgery for left-sided valvular heart disease have improved survival rates compared with patients with untreated TR post-open heart surgery (82.02% survival left and right heart valvular intervention at time of open-heart surgery vs 71.00% survival with left sided valvular intervention only; *P =* 0.048).^14^ In another analysis by Fujisawa et al^15^ found that TR severity at atrial fibrillation diagnosis in 2,211 Japanese patients was an associated predictor of subsequent hospitalization for heart failure. These findings suggest the need on the development of transcatheter TV interventions to treat this patient population commonly turned down for open-heart surgery.^14^^,^^16^, ^17^, ^18^

## Etiology

TR can manifest in a wide range of phenotypes with various mechanisms and etiologies. The European Tricuspid Focus Group has revised the classification of TR, subdividing it into primary TR, atrial secondary TR, ventricular secondary TR, and cardiac implantable electronic device (CIED)-related TR.^17^ Primary or organic TR results from anatomic abnormalities of the TV apparatus and includes subsets such as myxomatous degeneration, congenital malformations, traumatic lesions, rheumatic heart disease, and endocarditis. Secondary TR is attributed to annular dilation caused by right atrial (RA) or right ventricular (RV) enlargement and dysfunction from pressure or volume overload. Secondary TR may be associated with pulmonary hypertension caused by left-sided heart disease or atrial fibrillation with normal RV pressures. Secondary TR may also develop after left-sided valve surgery, sometimes unmasked in the setting of postoperative stunning, silent ischemic RV changes, or progressive RV negative remodeling from prior undetected clinically significant right sided valvular disease.^16^ Clinically significant TR can exist in the presence or absence of preserved left ventricular function, which adds to the complexity of phenotyping right-sided heart failure.^18^ CIED-related TR is a distinct entity with anatomical interruption of normal tricuspid leaflet coaptation planes resulting in progressive TR over time, and potential right sided heart failure.^19^^,^^20^

The understanding of TR etiology across APAC requires an understanding of socioeconomic determinants of health and disease pathophysiology prevalence according to country and type of health care setting (Central Illustration). Although the prevalence of rheumatic heart disease has declined in transitional countries,^21^^,^^22^ it remains a significant issue in some APAC nations. In the 2015 Global Burden of Disease Study, Watkins et al^23^ reported over 70% of the globe’s rheumatic heart disease were in the countries of India (13.17 million cases), China (7.07 million), and Pakistan (2.25 million).^21^ Chan et al^21^ additionally reported rheumatic heart disease as a significant social-economic disparity amongst under-represented communities such as the Maori and Pacific Islanders, and the Indigenous Australians (prevalence up to 15 per 1,000 in the Top End of the Northern Territory). The mechanism of rheumatic heart-related TR is complex. It can happen secondary to left heart disease (ie, rheumatic mitral/aortic valve disease), as pulmonary hypertension associated with rheumatic heart disease itself, or rarely, rheumatic involvement of TV. Moreover, socioeconomic disparity in access to care, and genetic determinants predisposing to repeated infections, yield to increased risk of repeated infection and progressive rheumatic heart disease and right sided heart failure.^24^ Additional risk factors for TR include long-standing atrial fibrillation.^25^ Patlolla et al^25^ demonstrated that in a population-based cohort of 232 patients with atrial fibrillation, nearly one-third developed moderate or greater TR over time. Prapan et al^26^ also found that significant TR in patients with atrial fibrillation was common and independently linked to adverse outcomes. In the Asia Pacific Heart Rhythm Society’s 2021 practice guidance on atrial fibrillation screening publication, Chan et al^21^ reported a 20-fold increased prevalence of atrial fibrillation in China over a period of 11 years and estimated a doubling of atrial fibrillation prevalence in the European Union by 2060. They also identified heterogeneity in practice patterns across different Asia Pacific countries in screening and detection of atrial fibrillation. Absence of a uniform model for screening for atrial fibrillation and for tricuspid valve disease in a population based, community based, or workplace-based environment limits a clear pathway for diagnosis and management of symptomatic TV disease.^21^

## Anatomy and Imaging Evaluation of TV

The TV is the largest cardiac valve with a normal TV annular area between 7 and 9 cm^2^.^27^ In general, the anterior leaflet is the largest scallop, the septal leaflet has the smallest radial length, and the posterior leaflet may have multiple scallops with overall smallest annular circumference. The anterior papillary muscle is vital because it supplies chordae to the anterior and posterior leaflets. The posterior papillary muscle provides chordae to the posterior and septal leaflets, and the septal papillary muscle may be absent. The tricuspid annulus has a complex 3-dimensional structure ∼20% larger than the mitral annulus.^28^ To aid procedure planning and execution, Hahn et al^29^ proposed a standardized nomenclature and classification of the TV into 6 types/subtypes based on echocardiogram: type I, 3 leaflets; type II, 2 leaflets; type IIIA, 4 leaflets with 2 anterior; type IIIB, 4 leaflets with 2 posterior; type IIIC, 4 leaflets with 2 septal; and type IV, >4 leaflets. Among these, type I is the most common seen morphology (53.9%), followed by type IIIB (32.1%).^29^

**Figure 1:**
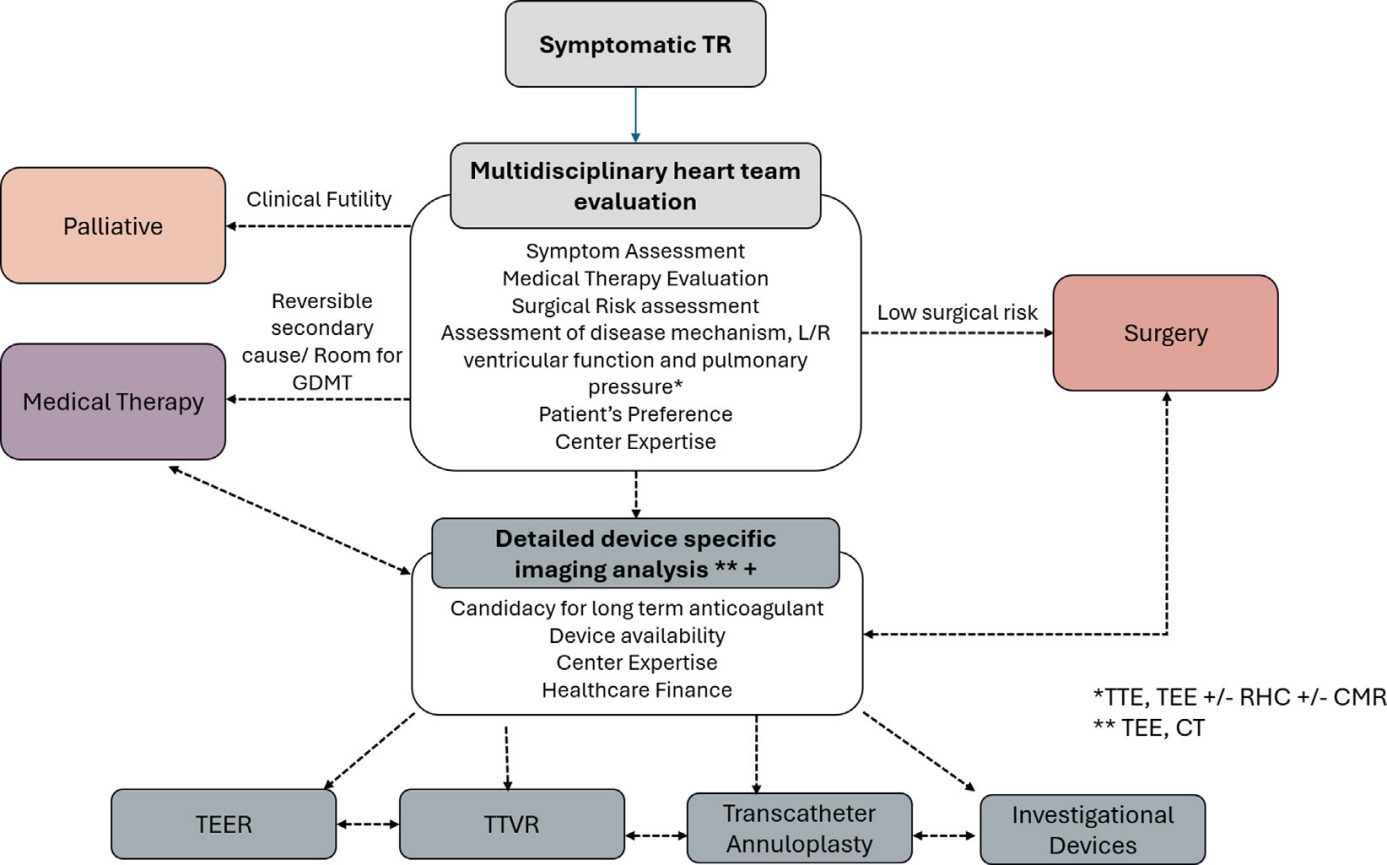
Algorithm for Evaluating Symptomatic Tricuspid Regurgitation Patients Proposed multidisciplinary heart team considerations when approaching clinical evaluation of a patient with severe symptomatic tricuspid regurgitation incorporate multimodality imaging physician expertise, surgical candidacy considerations, goal-directed medical therapy optimization, and anatomical evaluation for potential transcatheter tricuspid interventions. CMR = cardiac magnetic resonance imaging; CT = computed tomography; GDMT = guideline-directed medical therapy; L = left; R = right; RHC = right heart catheterization; TEE = transesophageal echocardiogram; TEER = transcatheter edge-to-edge repair; TTE = transthoracic echocardiogram; TTVR = transcatheter tricuspid valve replacement.

**Figure 2:**
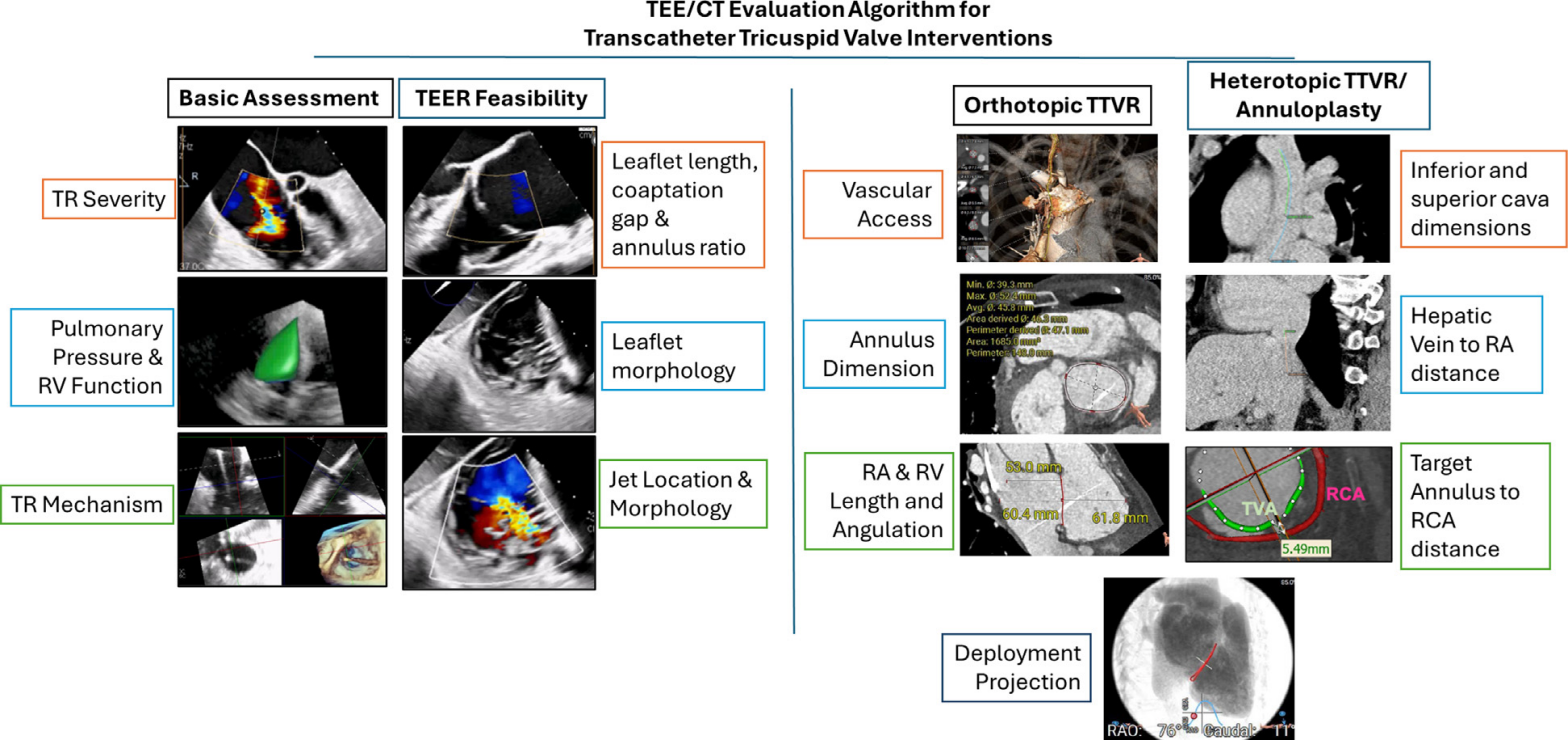
Imaging Evaluation Algorithm for Transcatheter Tricuspid Valve Interventions Multi-pronged multimodality imaging analysis is required for transcatheter tricuspid interventions. Multimodality imaging assists in anticipating and optimizing case-procedural planning to help the implanting team anticipate and overcome potential anatomical challenges. CT = computed tomography; RA = right atrium; RCA = right coronary artery; RV = right ventricle; TR = tricuspid regurgitation; other abbreviations as in Figure 1.

**Figure 3:**
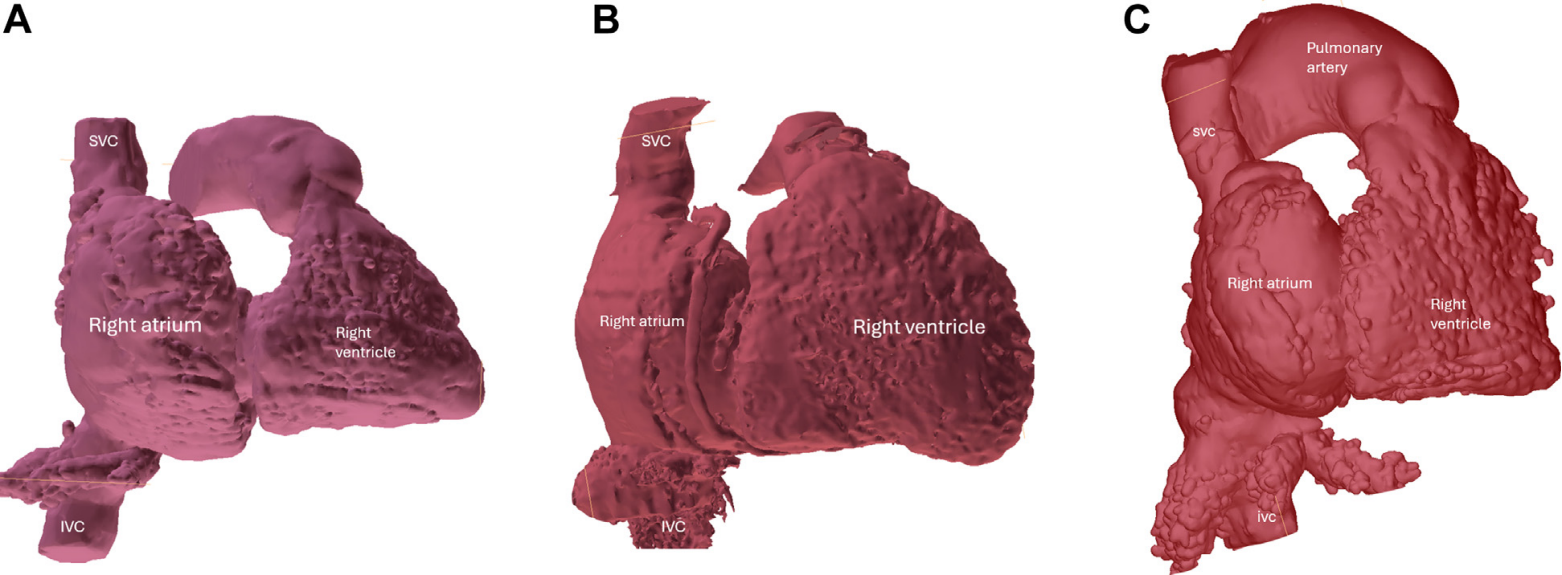
Computed Tomography 3-Dimensional Reconstruction Showing Atrial vs Ventricular Secondary Tricuspid Regurgitation (A) Atrial secondary tricuspid regurgitation; (B) ventricular secondary tricuspid regurgitation. (C) Overlapping features of atrial and ventricular secondary tricuspid regurgitation with basal enlargement of the right ventricle with right atrial enlargement. IVC = inferior vena cava; SVC superior vena cava.

With increasing interest and need for TV intervention, detailed transthoracic and transesophageal echocardiographic (TEE) assessments are crucial in the evaluation of TR and feasibility of transcatheter TV intervention (Figure 1). It is important to quantify the TR severity, the TV leaflet morphology, and the mechanism of TR (Figure 2). Two- and 3-dimensional assessments are needed to identify any abnormality of the components of the TV apparatus (tricuspid leaflets, chordae, papillary muscles, or annulus) from congenital or acquired causes.^30^ If no organic cause is identified, it is important to assess for secondary causes of TR, further classifying the pathophysiology into atrial secondary or ventricular secondary TR.^6^ In atrial secondary TR, the main disease mechanism is leaflet malcoaptation caused by a significant dilation of the tricuspid annulus secondary to RA enlargement most seen in patient with long-standing atrial fibrillation or heart failure with preserved ejection fraction.^31^ In ventricular secondary TR, progressive enlargement of the RV leads to TV leaflet tethering and annular dilation with associated regurgitation. As opposed to ventricular secondary TR, in atrial secondary TR, there is trivial TV leaflet tethering, disproportionate RA to RV enlargement, RV conical re-modelling with predominant enlargement of RV basal dimension (Figures 3A and 3B), and usually a preserved left and right ventricular function when evaluated during sinus rhythm or rate controlled atrial fibrillation.^31^ However, it is not uncommon for patients to have overlapping features of atrial and ventricular secondary TR (Figure 3C).^31^ In patients with CIED-related TR, the echocardiographic assessment is anatomically challenging to image secondary to the complex mechanism and imaging shadowing from the CIED leads. CIED-related TR may be secondary to mechanical interaction of CIED lead and the TV (ie, TV leaflet impingement, leaflet or chordal entanglement, chordal rupture, leaflet adherence, or leaflet laceration or perforation), or secondary to pacing-related TR.^20^^,^^32^ Finally, assessment of RV function and pulmonary pressure are also important not only to determine the mechanism of TR but also for staging the presence, absence, and degree of RV dysfunction (Figures 1 and 2). In most clinical trials, echo-derived calculated pulmonary arterial systolic pressures >70 mm Hg trigger further invasive testing to evaluate for the presence of undetected pulmonary hypertension. A dedicated right heart catheterization is performed to assess the mean pulmonary pressure, wedge pressure, and pulmonary vascular resistance to help predict patient’s outcome after TV interventions.^33^ Assessment of left and right ventricular functions is also important. In most tricuspid clinical trials, patients with a low left ventricular ejection fraction (<30%) are either excluded or are treated maximally with guideline-directed medical therapy, transcatheter edge-to-edge repair, cardiac resynchronization therapy, and so on, before considering TR interventions. Patients with severe RV dysfunction might be more prone to afterload mismatch after TV intervention, especially transcatheter tricuspid valve replacement (TTVR), which usually eliminates TR. Therefore, prehabilitation and postprocedural optimization of RV dysfunction would be important.

TEE is necessary to help multidisciplinary heart teams screen patient’s anatomy for potential TEER and replacement technologies (Figure 2). With the current understanding and clinical experience, the TV anatomy can be classified into favorable, feasible, or extremely challenging category for TEER. Favorable anatomy includes those with confined prolapse/flail segment, a coaptation gap of <7 mm, central or anteroseptal jet origin, a leaflet-annular ratio of ≥1.06, and 2- or 3-leaflet morphology. Feasible anatomy includes those with a coaptation gap of 7 to 10 mm,^34^^,^^35^ posteroseptal jet origin, a leaflet-annular ratio of <1.06,^36^ 4-leaflet morphology,^37^ or presence of incidental CIED lead with no leaflet impingement or adhesion. Whereas extremely challenging anatomy includes a coaptation gap *>*10 mm, TR jet originate from anteroposterior commissure,^38^ rheumatic tricuspid leaflet change (leaflet retraction, calcification or sub-valvular apparatus calcification), leaflet perforation, marked leaflet tethering, or presence of CIED lead with leaflet impingement/adhesion. Recently, Gercek et al^39^ further defined the GLIDE score (Gap ≥6 mm, Location of jet at posteroseptal/anteroposterior/diffuse, Image quality limited, Dense chordal structure, En face TR morphology star-shaped) to help predict acute procedural success in tricuspid-TEER. Here, the authors found TR reduction ≥2 grades and ≤ moderate residual TR could be achieved in >90% of patients with a preprocedural GLIDE score 0-1, but only in 5.6% and 16.7% of those with GLIDE score ≥4.^39^

Preprocedure multidetector retrospectively acquired ECG gated contrast computed tomography (CT) scans are of additive value for planning transcatheter tricuspid interventions (eg, TTVR or annuloplasty) (Figure 2). The right heart CT assessment allows for device specific patient anatomical planning. Multidetector contrast-enhanced retrospectively gated CT studies are commonly used to assess to patient anatomical device eligibility including but not limited to the following (Figure 2): 1) vascular access for device delivery; 2) TV annular dimension if orthotopic TTVR is planned; 3) RA and RV length for delivery system entry and their angulation for device steering; 4) deployment projection; 5) inferior vena cava, superior vena cava dimensions and hepatic distance if heterotopic valve is planned; and 6) the location of the right coronary artery with respect to tricuspid annulus for any annuloplasty interventions.

## Presentation and Evaluation

Patients with significant degree of tricuspid regurgitation may remain undetected for an extended period, leading to progressive RV dilation and dysfunction.^40^ In left-sided valve diseases such as mitral regurgitation and aortic stenosis, elevated left atrial pressure and postcapillary pulmonary hypertension manifest as symptoms like exertional dyspnea. Conversely, in TR, elevated RA pressure is transmitted to the venous system of the entire body, leading to relatively insidious symptom of poor cardiac output and poor functional capacity.^41^ Patients with TR typically do not present with an overt cardiac murmur^42^ and are frequently devoid of classical left-sided heart failure signs and symptoms even when moderate-severe TR is incidentally detected on screening echocardiographic examinations. However, if patients exhibit clinically overt symptoms and signs of right HF, the TR is often much more advanced and likely found with concomitant negative RV remodeling.^43^ Similar to European and North American registry reporting, TR patients in the APAC nations often present late and experience delayed referral for specialist assessment and consideration of surgical or percutaneous intervention.^44^ As a result, a significant number of patients in the APAC regions present with large TV coaptation gap and advanced RV failure, making TEER anatomically challenging with uncertain long-term durability (Central Illustration). Current guidelines suggest treatment of primary TR should occur before signs of end-organ damage manifest and before significant RV dysfunction arise.^45^ The latest European valvular heart disease guidelines recommend that interventional treatment of secondary tricuspid regurgitation may be considered in experienced Heart Valve Centers for symptomatic but inoperable patients who are anatomically eligible and have the potential for a clinical benefit from the procedure.^17^

A comprehensive assessment is recommended in treating symptomatic TR patients (Figure 1). A multidisciplinary heart team with a structural heart interventionalist, interventional imaging physician, cardiac surgeon, heart failure specialist, electrophysiologist, cardiac anesthetist, intensivist, and cardiac advanced practice providers is essential to determine the appropriate treatment strategy and optimize the perioperative care in TR patients. To guide the appropriate treatment strategy, the multidisciplinary heart team needs to assess the patient’s symptoms, goal-directed medical therapy, surgical risk, disease mechanism, left and right cardiac function, and pulmonary pressure (by transthoracic echocardiogram or TTE and right heart catheterization in borderline cases). Right heart catheterization is important in borderline pulmonary hypertension cases because it helps to delineate the mechanism of TR, guide treatment feasibility and inform the prognosis after TV intervention.^46^

In the APAC nations, care pathways need to be developed with respect to an understanding of cultural and socioeconomic determinants of health specific to each country (Figure 1). It is important to consider the patient’s preference and local health care financial burden in making a treatment decision. In patients with end-stage heart failure who are deemed medically futile for invasive therapy by the multidisciplinary heart team, a palliative care approach may be considered. In patients with reversible secondary causes of TR (eg, left heart disease, primary pulmonary hypertension), treatment of the primary disease should be considered. Upon first detection of symptomatic severe TR, patients should start on diuretic therapy. If patients have refractory symptoms despite optimization of diuretic therapy, in low surgical-risk patients, surgery can be considered. In a high surgical-risk cohort, transcatheter TV intervention may be considered based on each patient’s anatomic suitability and the local health center’s level of expertise. Detailed TEE imaging together with preprocedural multidetector contrast-enhanced CT assessment will help guide patient-specific transcatheter options (Figure 2). Cardiac magnetic resonance imaging is the current gold standard method for quantitation of RV size and systolic function, and should be considered, if available, to assess the baseline RV function and postoperative remodeling in patients receiving tricuspid interventions. The multidisciplinary heart team will need to account for the patient’s ability to tolerate long-term anticoagulation, national and local health system access to transcatheter device technologies, the local health center’s transcatheter and imaging expertise, and local health care and socioeconomics as they pertain to the patient and the hospital’s ability to access the new technologies when making treatment decisions (Figure 1). Finally, when patients may not anatomically meet the criteria for commercially available transcatheter technologies, national and local level access to new technologies via clinical trials may bring new hope for patients without other treatment options.

## Interventions

### Medical therapy

Current North American and European guidelines recommend a trial of diuretic agents (Class IIa) for symptomatic TR.^17^^,^^45^ The intent of diuretic usage is aimed at decreasing RV and RA volume overload to help relieve systemic congestion. With the rapidly evolving transcatheter TV intervention options, there is a paucity of data on optimal guideline-directed medical therapy regimens for right heart failure and optimal diuretic dosing before consideration of TV intervention. Additionally, evolution of new heart failure medications such as sodium glucose cotransporter 2 inhibitors have gained much attention because of their positive effect in heart failure patients with preserved ejection fraction. Current ongoing trials evaluating for potential impact of sodium glucose cotransporter 2 inhibitors on RV function, progression of TR, heart failure hospitalization, and cardiovascular endpoints (Reduction-TR [SGLT2 Inhibitor for Severe Tricuspid Regurgitation; NCT05686616], PROVE [Pharmacological Reduction of Right Ventricular Enlargement; NCT04345796], EVENT [Enavogliflozin Outcome Trial in Functional Tricuspid Regurgitation; NCT06027307]) may provide more promise to identifying optimal goal-directed medical therapy regimens for the treatment of TR.

### Surgery

In the 2021 European Society of Cardiology/European Association for Cardio-Thoracic Surgery guidelines,^17^^,^^45^ TV surgery is recommended in patients with severe TR undergoing left-sided valve surgery, primary or secondary (Class I), or TV annular dilatation (>4 cm, Class 2a), symptomatic patients with isolated severe primary TR (Class 2a), and symptomatic patients with isolated severe secondary TR without pulmonary hypertension or left heart disease (Class 2a). A similar recommendation was given in the Japanese valvular heart disease guidelines.^47^ In a U.S. National Inpatient Sample study of patients hospitalized for surgical tricuspid valve repair or replacement, trends for postsurgical outcomes were identified based on type of tricuspid surgical intervention.^48^ Isolated surgical TV replacement was associated with the highest in-hospital mortality (10.9%) compared with surgical repair (5.9%) and associated with higher rates of permanent pacemaker implantation (35.0% vs 13.4%). Differences in surgical tricuspid replacement vs surgical tricuspid repair mortality rates were thought to be multifactorial, from surgeons potentially working with a sicker RV cohort of patients with greater annular dilatation where surgeons felt a replacement device would have greater durability than a repair strategy, to an overall sicker patient population. In the APAC region, similar trends in tricuspid surgery exist. In the China DVD study, only 3.2% of patients with right-sided valvular heart disease, mainly TR, received surgical treatment, much less than those with left-sided valvular heart disease.^9^ In Japan, only 16.4% of patients received TV surgery, much less than those with aortic stenosis (53.6%) and mitral regurgitation (38.3%).^11^ The majority were TV repair (12,342 of 12,647 in 2015-2016 and 11,469 of 11,869 in 2013-2014), compared with TV replacement (305 of 12,647 in 2015-2016 and 359 of 11,869 in 2013-2014).^49^ Tricuspid surgical intervention data in itself is a heterogenous data set.

Several surgical repair techniques have been described. Most surgical registry data collecting tricuspid surgical outcomes do not distinguish between types of tricuspid repair strategies, ie, ring annuloplasty, Kay suture annuloplasty, De Vagas suture annuloplasty, triangular resection, or double-orifice valve repair. Combinations of surgical bicuspidization and annuloplasty have additionally been reported to have durable outcomes at reducing TR severity on 3-year follow-up.^50^ As the field of tricuspid interventions develops, surgical and transcatheter techniques will continue to evolve.

Unlike the Western world, there are wide disparities in access to cardiac surgery in APAC countries caused by differences in socioeconomic status.^51^^,^^52^ In less developed South Asian countries, despite having more than 6 times the total population, these nations combined performed less than one-half of the annual number of cardiac surgeries performed in the United States.^51^ On the other hand, in more developed regions like Japan, cardiac surgeries are not uncommonly performed.^52^ Besides, most tricuspid valve surgeries are performed as part of a multivalvular surgery, most commonly tricuspid annuloplasty during mitral valve surgery in APAC.^52^ Tricuspid annuloplasty during multivalvular surgery is a fundamental skill for cardiac surgeons in APAC,^53^^,^^54^ although isolated tricuspid valve surgery was rarely performed.^52^

### Transcatheter interventions

**Figure 4:**
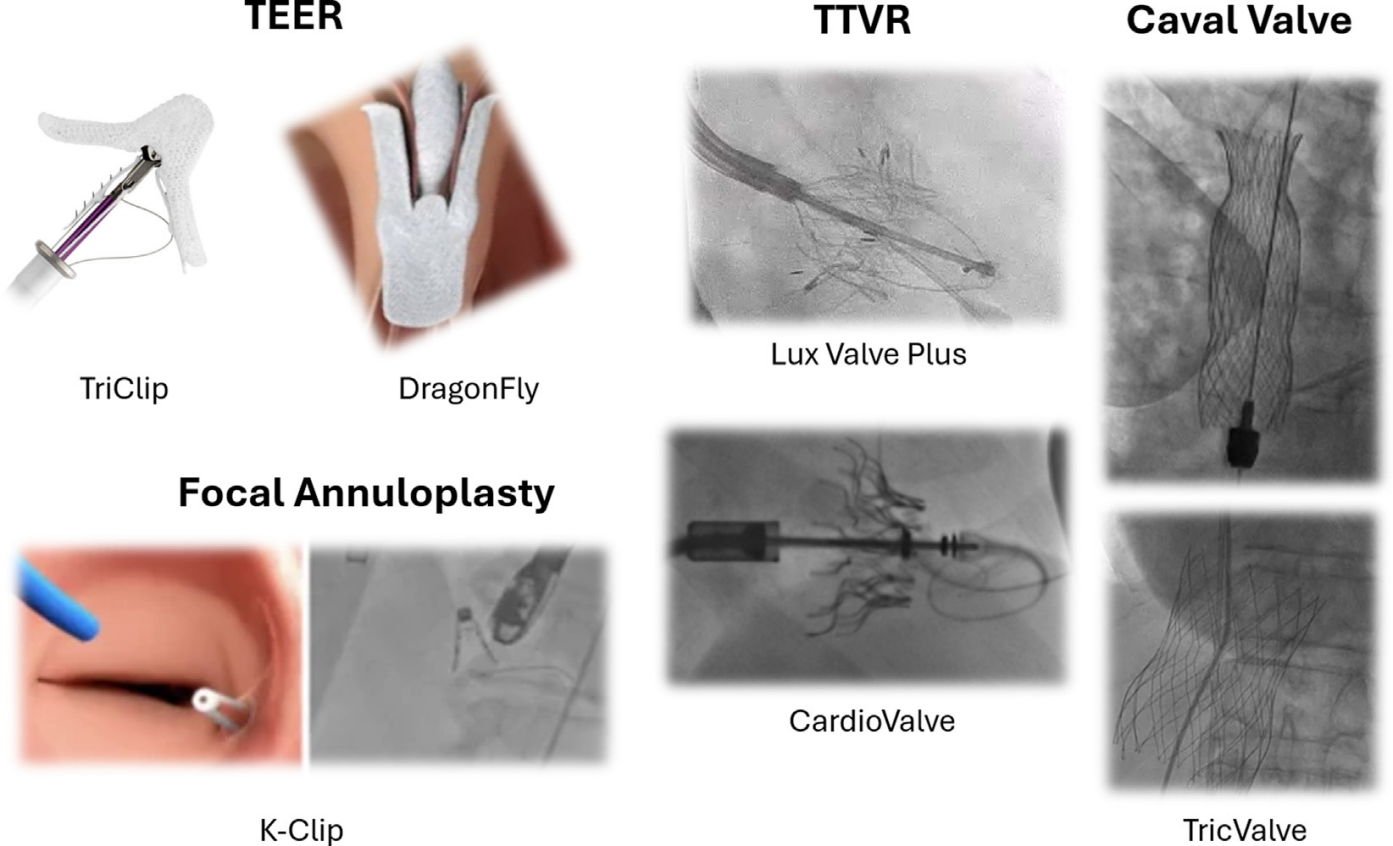
Overview of Current Transcatheter Tricuspid Valve Devices in Asia Pacific Current TEER, TTVR, caval valve, and annuloplasty technologies currently available in commercial or investigational trial within Asia Pacific region are depicted. Abbreviations as in Figure 1.

Several types of transcatheter TV therapies are commercially available or under development, including transcatheter repair and replacement devices (Figure 4). Transcatheter repair therapies include leaflet approximation devices, annuloplasty, and other devices such as “spacers” (which fill the coaptation gap). Transcatheter replacement devices include heterotopic and orthotopic valve replacement. However, device options to treat TR are limited in APAC for several reasons: 1) significant difference in device regulatory process and device reimbursement policy between different countries/regions; 2) lower market priority in APAC by major cardiovascular device companies, which defers the launch of new devices in APAC compared with the United State and Europe and excludes the access to investigational devices in APAC; and 3) lack of domestic device innovation until more recently more novel devices are originated from APAC countries. Currently, TEER using the TriClip is commercially approved in limited locations in APAC (including but not limited to Hong Kong, Taiwan, Thailand, Malaysia, Singapore), while more TEER TR devices are under clinical investigation (Table 1). We will therefore focus on reviewing the features and evidence of devices that are commercially available or have been in investigational device usage in the APAC region (Figure 4) (including TEER devices [TriClip (Abbott) and DragonFly (Valgen Medtech)], TTVR devices [Lux Valve Plus (Jenscare), CardioValve (Venus Medtech), and TricValve (Products + Features)], and focal annuloplasty device K-Clip [Huihe Medical]).

**Table 1:** Transcatheter Tricuspid Devices Available in Asian Countries

	Mainland China	Hong Kong SAR	Taiwan	Japan	South Korea	Australia/New Zealand	India	Thailand	Singapore	Malaysia
TriClip	a	**√**	**√**			**√**		**√**	**√**	**√**
DragonFly	b									
K-Clip	b	c								
Lux Valve Plus	b	c	d			c		d	d	
CardioValve		c								
TricValve	b	**√**	c			c	**√**	**√**	**√**	**√**

a Will be available likely within year 2024 in limited area.

b Under clinical research.

c Compassionate use only.

d Will be available for compassionate use within year 2024.

### Tricuspid TEER

To date, tricuspid TEER remains the most common transcatheter TV repair procedure in the APAC region. Currently, 2 devices (TriClip, Abbott; PASCAL Precision, Edwards Lifesciences) have received commercial use approval in Europe, and the TriClip device is also commercially available in the United States. In TEER therapy, TR is tackled by leaflet approximation at the regurgitation site to achieve improvement in leaflet coaptation and decrease of regurgitant orifice opening. The dedicated TEER system (TriClip, Abbott Vascular) with a shorter guiding catheter curvature and steerable plane of motion (septal-lateral plane) was developed to facilitate co-axial TV access. TriClip (Abbot Vascular) was recently launched in limited sites among designated APAC nations together with the fourth-generation TEER system (Table 1). The fourth-generation devices have 4 different clips length and widths (XTW, XT, NTW, and NT) with controlled gripper actuation to permit optimized independent leaflet grasping. The efficacy and safety of Tricuspid TEER devices have been confirmed by single-arm studies^55^ and a randomized controlled trial (TRILUMINATE).^56^ The TRILUMINATE (TRILUMINATE Study With Abbott Transcatheter Clip Repair System in Patients With Moderate or Greater TR) pivotal trial showed that the incidence of death or TV surgery and the rate of hospitalization for heart failure did not appear to differ between groups at 1 year follow-up. However, the quality of life, as measured by the Kansas City Cardiomyopathy Questionnaire score, was improved in the TEER group, with a mean of 12.3 ± 1.8 points increase in the TEER group compared with 0.6 ± 1.8 points in the control group (P < 0.001).55 Besides, the bRIGHT (An Observational Real-world Study Evaluating Severe Tricuspid Regurgitation Patients Treated With the Abbott TriClip Device; NCT04483089) real-world registry also showed that patients received TriClip therapy had significant improvements in NYHA functional class (21% to 75% I/II; P < 0.0001) and a mean 19 ± 26-point improvement in Kansas City Cardiomyopathy Questionnaire score (P < 0.0001) at 1 year compared with baseline.^57^ Furthermore, the latest Tri.Fr randomized controlled trial showed that among patients with ≥ severe symptomatic TR, tricuspid TEER with TriClip plus guideline-directed medical therapy was superior to guideline-directed medical therapy alone in achieving an improvement in the clinical composite score at 12 months (74.1% vs 40.6%, effect estimate was 0.67, 95% CI: 0.61-0.72; *P* < 0.0001), which included improvement in NYHA functional class, quality of life assessment using the Patient Global Assessment of health, and the incidence of major adverse cardiovascular events. The positive outcome was mainly driven by the improvement in symptom classification and quality of life assessment, without a significant difference in incidence of major adverse cardiovascular events and cardiovascular mortality. With the background of either normal left ventricular systolic function or impaired left ventricular systolic function treated maximally with guideline-directed medical therapy before enrollment, a longer follow-up might be needed to assess these hard clinical endpoints. Moreover, the study also reaffirmed that tricuspid TEER was associated with low incidence of in-hospital complications (8%), and in-hospital mortality was 0.6%.^58^ TriClip received U.S. Food and Drug Administration approval in April 2024. The PASCAL device and delivery system is a nitinol-based device with a passive closing mechanism. It can be used for both mitral and tricuspid TEER, enabling combined procedures. Unlike the TriClip system, PASCAL has a central spacer where the arms clasp the leaflets to reduce TR.^59^ The device has a unique elongation feature that minimizes the risk of leaflet entanglement. Two sizes are available, and continuous pressure monitoring is integrated into the steerable catheter. Early experience with the PASCAL system showed sustained 1-year TR reduction to moderate or less in 86% of 30 patients.^60^ The ongoing CLASP TR pivotal trial is randomizing patients between optimal guideline-directed medical therapy and TEER with the PASCAL system, with a 2-year endpoint. Among the APAC nations, the PASCAL device is now commercially available in Australia and Japan for treating mitral regurgitation, but not TR. The Dragonfly system^61^ is a TEER device developed in China. The device has a central, compressible filler that can be compressed to different angles with the clip arm locking at 0° to 45° to fill the residual regurgitant orifice, potentially reducing leaflet tension. The device has 4 different sizes. Dragonfly is approved in China to treat mitral regurgitation and is currently under investigational clinical trial for TR.

#### Step-by-step TEER

**Figure 5:**
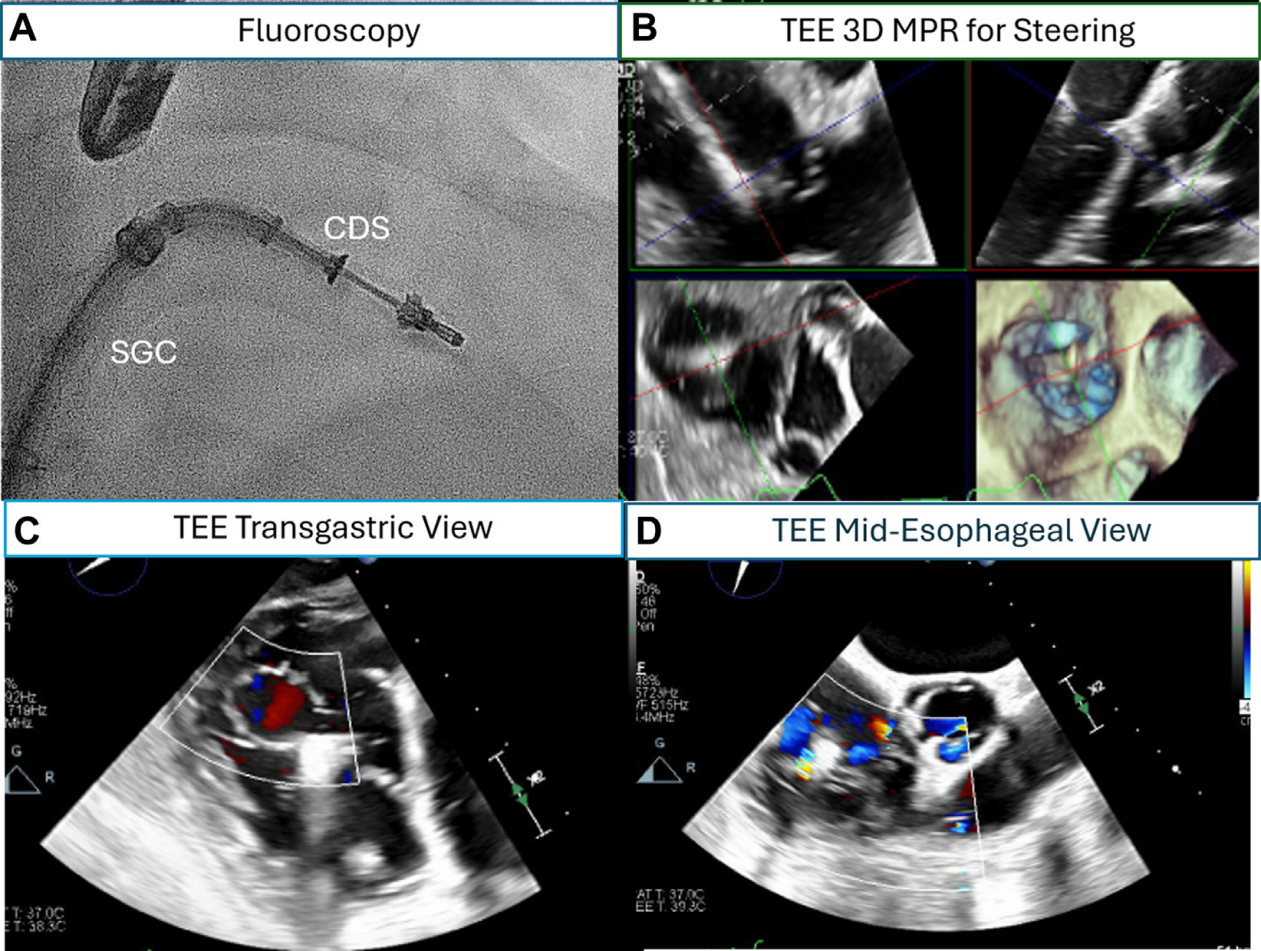
Intraprocedural Images of a Tricuspid-Transcatheter-Edge-to-Edge Repair Using the TriClip Device (A) Fluoroscopy of a TriClip Steerable Guide Catheter (SGC) and Clip Deliver (CDS) System. (B) Transesophageal echocardiogram (TEE) 3-dimensional (3D) multiplanar reconstruction (MPR) to guide the steering of TriClip system. (C) TEE transgastric view to confirm the clip arm orientation and tissue bridge after clip closure. (D) TEE midesophageal view to assess final tricuspid regurgitation after clip implantation.

The TEER procedure is performed via femoral venous access under fluoroscopy and TEE guidance and mostly under general anesthesia (Figure 5). The delivery sheath steering aims at aligning the clip perpendicular to the line of coaptation at the target zone under interventional imaging physician led-TEE guidance. In cases of large coaptation gaps, additional techniques, including diuresis, Valsalva maneuver, use of positive end expiratory pressure,^62^ or a standardized Trendelenburg tilting of the interventional table by 10°,^63^ have been described to reduce the coaptation gap to facilitate leaflet grasping. Additional approaches to TEER, such as incorporating the “zipping” technique, in which multiple clips are used alongside the first implant starting with a more commissure grasp to zip along the leaflet coaptation plane to facilitate subsequent clipping, have been effective in reducing TR.^64^^,^^65^ The greatest increase in cardiac output may be obtained with anterior- and posterior-septal leaflet tip grasping because of the increased leaflet coaptation area of these specific leaflet combinations.

### Annuloplasty

Potential advantages of annuloplasty include the preservation of leaflet anatomy and its role as an adjunctive procedure to other interventions (eg, annuloplasty combined with TEER). The Cardioband system (Edwards Lifesciences), analogous to the surgical ring is currently CE-mark (Conformite Europeenne) approved.^66^ Cardioband is implanted through the femoral vein under interventional imaging physician led-TEE guidance with multiple anchoring screws to achieve reductions in tricuspid annular size and TR. In a multicenter early feasibility study of 37 patients, procedural success was 83%, and at 1-year follow-up, 73.1% of patients experienced a ≥2-grade reduction in TR, with 73.0% having moderate or less residual TR.^67^ Additionally, the proportion of patients in NYHA functional class I/II doubled from baseline to 1 year, and there was a clinically significant improvement in the Kansas City Cardiomyopathy Questionnaire score (19.0 points).^67^ The TRI-REPAIR (TrIcuspid Regurgitation RePaIr With CaRdioband Transcatheter System) observational study enrolled 30 patients with symptomatic functional TR.^68^ At 2-year follow-up, echocardiography showed a 16% reduction in septolateral annular diameter, and ≤2+ TR in 72% of patients.^68^ The 6-minute walking distance and Kansas City Cardiomyopathy Questionnaire score improved by 73 m and 14 points, respectively.^68^ However, because of the complexity in implantation with long procedure and fluoroscopy time required,^69^^,^^70^ the device is only limited to a few experienced centers.

**Figure 6:**
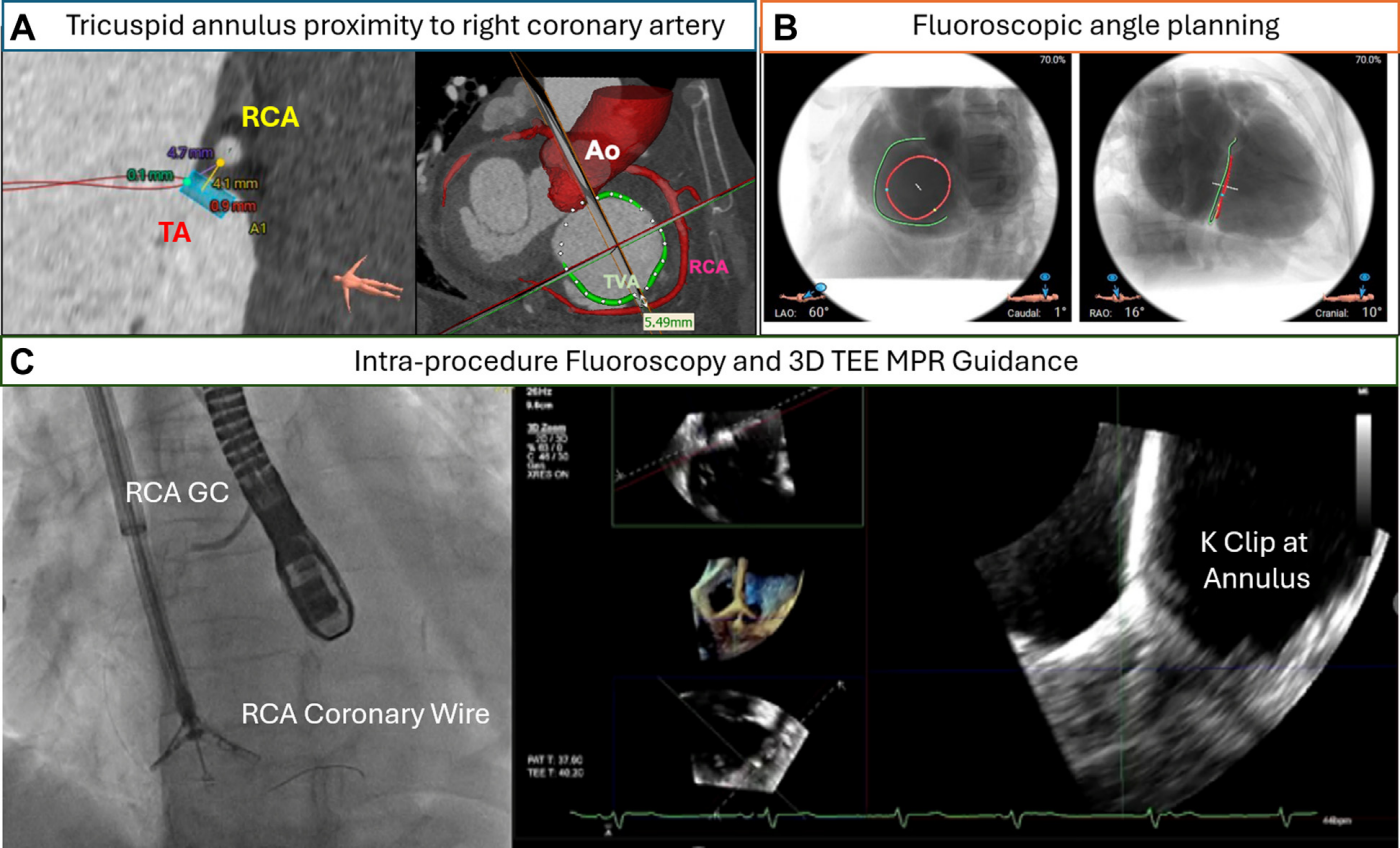
Multimodality Imaging to Guide K-Clip Transcatheter Annuloplasty (A) Preoperative computed tomography was performed to assess the tricuspid annulus (TA) dimension and the proximity of right coronary artery (RCA) to the TA. (B) Fluoroscopic projection for device implantation was determined. (C) During the procedure, an RCA guide catheter (GC) with a coronary wire is inserted to look for any coronary impingement by the K-Clip, and 3D TEE MPR is used to guide the steering and implantation of K-Clip. Abbreviations as in Figure 5.

The K-Clip (Huihe Medical Technology) is a focal annuloplasty device delivered from the right internal jugular vein (Figure 6, Video 1). It achieves annular and TR reduction through a modified transcatheter tricuspid annular reduction pathway mimicking the Kay Bicuspidization surgery. The K-Clip delivery sheath aligns to the target tricuspid annulus commissure of interest and, using a proprietary corkscrew technology, captures and retracts the targeted portion of the tricuspid annulus within the K-Clip clamps to allow for clip closure, pseudo-bicuspidization, apposition, and reduction of the tricuspid annulus. The TRISTAR prospective registry of 39 TR patients demonstrated the K Clip was successfully implanted in all patients, with 1 to 3 devices deployed per patient, which resulted in 100% of patients achieving at least 1 grade TR reduction, and accompanied improvement in patient’s quality of life (6-minute walk increased by 78 m and Kansas City Cardiomyopathy Questionnaire score increased by 11 points) at 30 days^71^ (Table 1).

### Orthotopic TTVR in native tricuspid annulus

A dedicated TTVR device for native TR is now available in certain APAC regions. The Evoque system (Edwards Lifesciences), a self-expanding device using a mix of leaflet and annular fixation with a dedicated delivery system, is one of the first TTVR devices available to multidisciplinary heart teams for commercial use. It is approved for commercial clinical use in Europe and America, and under investigational trial usage in Japan, and will only be available for commercial use in limited APAC sites earliest in 2025. The device is delivered from femoral venous access and performed under general anesthesia and interventional imaging physician led intraoperative TEE guidance. The TRISCEND (Edwards EVOQUE Transcatheter Tricuspid Valve Replacement: Pivotal Clinical Investigation of Safety and Clinical Efficacy using a Novel Device) safety-efficacy trial enrolled 176 patients.^72^ The degree of tricuspid regurgitation was reduced to ≤ mild in over 97% of patients (*P <* 0.001), and NYHA functional class I or II was achieved in 93.3% of patients (*P <* 0.001). The 30-day cardiovascular mortality was 1.7% and 1-year all-cause mortality was 9.4%.^72^ TRISCEND II pivotal trial comparing Evoque TTVR with medical therapy has been completed and its favorable 6-month outcomes led to recent FDA approval.

**Figure 7:**
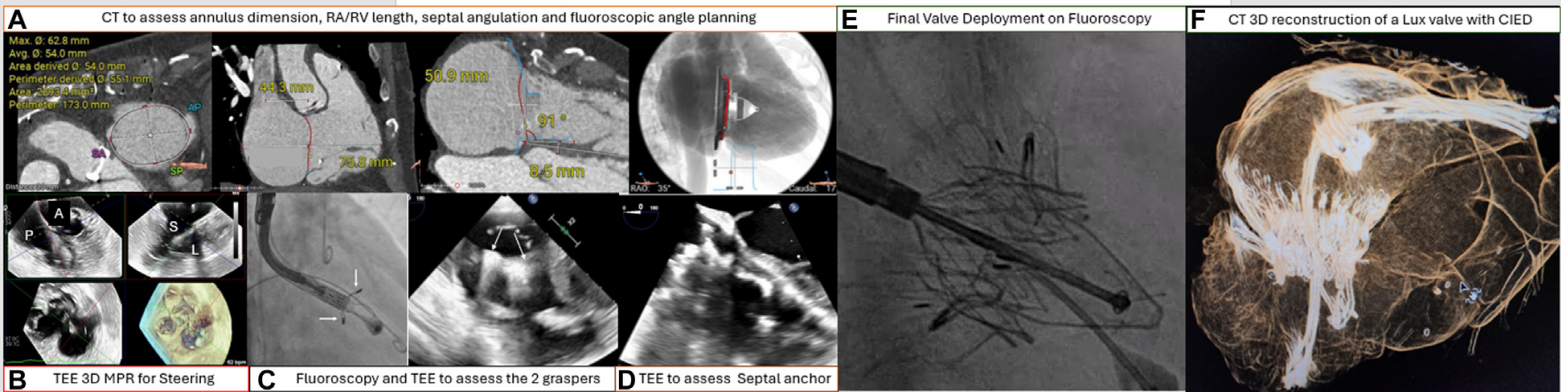
Multimodality Imaging to guide the LuX Transcatheter Tricuspid Valve Replacement (A) Preoperative computed tomography (CT) was performed to assess the tricuspid annulus size, right atrial (RA) and right ventricular (RV) length, interventricular septum angulation, and device fluoroscopic implantation projection. (B) TEE 3D MPR is used to guide the steering of delivery system. (C) Fluoroscopy and TEE STR used to assess the location of the 2 graspers (white arrows) of the LuX valve. (D) TEE at midesophageal view confirms contact of the septal anchor with the interventricular septum. (E) After septal anchor implantation, the LuX valve is deployed. (F) Follow-up imaging shows the supra-annular cuff of the LuX valve apposed to the right atrial wall to minimize any paravalvular leak, and the position of the septal anchor within the interventricular septum. Abbreviations as in Figure 5.

Several other TTVR devices are under clinical trial or approved for compassionate use in selected sites in the APAC Region (Table 1). The LuX valve (Jenscare) is a TTVR device developed from China. The device was first implanted using minimally invasive surgical techniques via a right mini-thoracotomy transatrial approach. The LuX valve has 3 mechanisms of anchoring: 2 graspers; 1 to capture the anterior leaflet and a second grasper to capture the posterior tricuspid leaflet; with an additional septal anchor deployed within the mid-interventricular septum for valve anchoring and stability. The LuX valve additionally has a unique design with a supra-annular right atrial cuff extending and abutting the RA walls to decrease paravalvular leakage. The second-generation LuX valve Plus system is delivered via right internal jugular access^73^^,^^74^ (Figure 7, Video 2). This device is currently undergoing clinical trial in Europe and investigational trial in China. The CardioValve (Cardiovalve Inc) is a TTVR system with an additional sealing cuff to minimize perivalvular leaks (Table 1). Other early-phase investigational devices that are not yet available in Asia include the Intrepid (Medtronic Inc), the VDyne (VDyne),^75^ the Topaz (TRiCares SAS),^76^ the TriSol Valve (TriSol Medical Ltd),^77^ and the Duo Valve (Croivalve).

### Heterotopic TTVR

**Figure 8:**
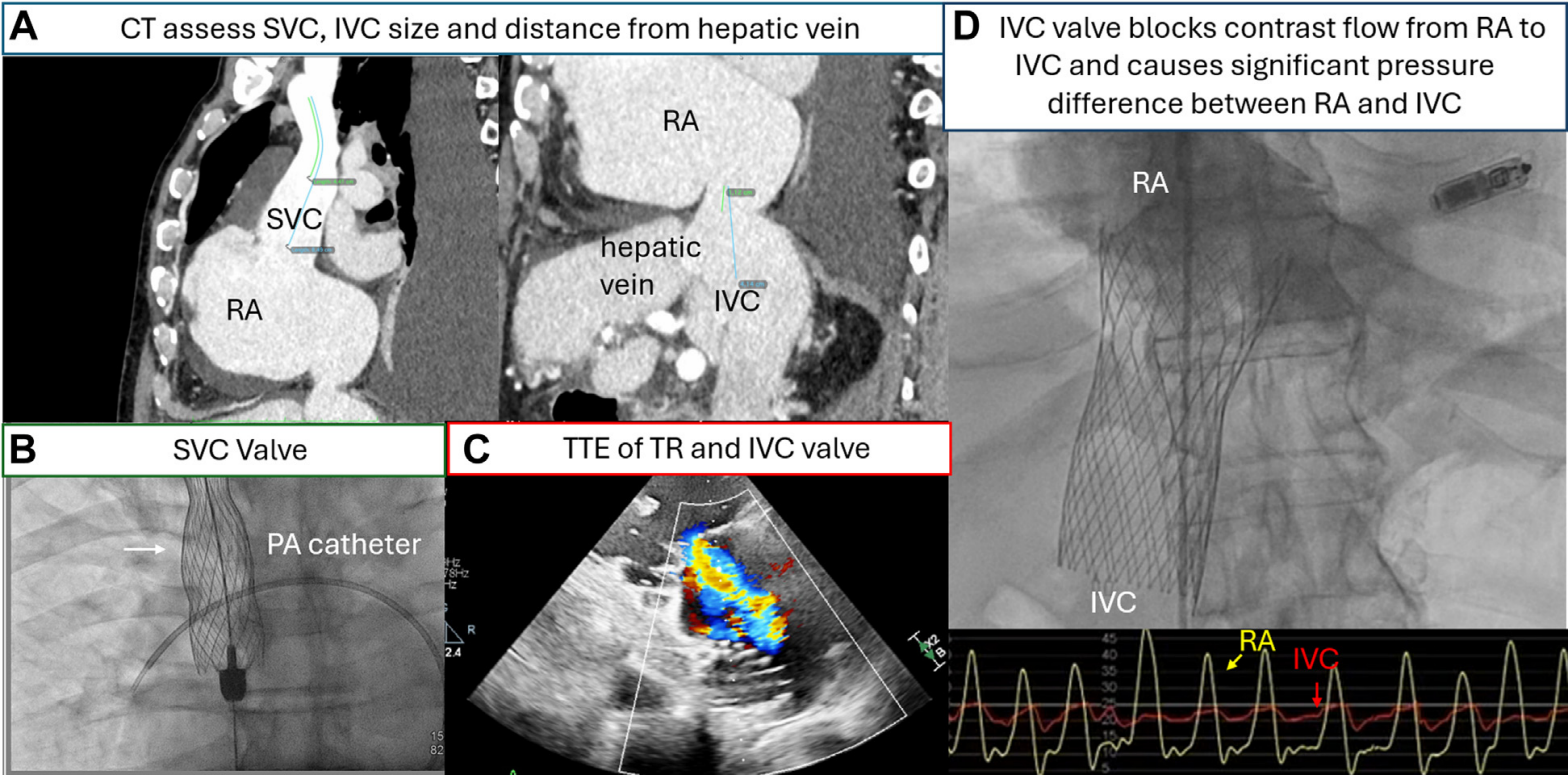
Multimodality Imaging to Guide TricValve Heterotopic Transcatheter Tricuspid Valve Replacement (A) Preoperative CT was performed to assess the sizes of SVC, IVC, and the distance of right atrium to the first hepatic vessel. (B) Implantation of the SVC valve with the belly (white arrow) of the valve positioned superior to the pulmonary artery additionally confirmed by a pulmonary artery (PA) catheter. (C) TTE showing the IVC valve in-situ with the presence of severe tricuspid regurgitation (TR). (D) The implanted IVC valve blocks contrast flow from right atrium (RA) to IVC and causes significant pressure gradient between RA and IVC. Abbreviaitons as in Figures 1 and 3.

Caval valve implantation has been used to protect organs from venous hypertension and reduce TR backflow primarily in patients with anatomical contraindications for conventional transcatheter interventions.^78^ TricValve (Products + Features) is a heterotopic bicaval valve delivered from femoral venous access (Figure 8, Video 3). The combined analysis of TRICUS/TRICUS EURO studies included 44 patients with severe symptomatic TR. At 1-year post-TricValve implantation follow-up, 95.5% of patients (42 of 44) patients, experienced either an increase in ≥15 points from baseline in Kansas City Cardiomyopathy Questionnaire score, improvement to NYHA functional class to I or II, or an increase ≥40 m in the 6-minute walk test.^79^ Patients who underwent TricValve implantation were also noted to have a reduction of the RV mid-diameter at 6-month follow-up CT from 48.6 ± 9.9 m to 43.0 ± 7.3 mm *(P =* 0.001).^80^ TricValve has 2 dedicated self-expanding nitinol stents with bovine pericardial leaflets implanted in the inferior vena cava and superior vena cava to abolish the backflow for systemic venous congestion. The implantation can be performed under pure fluoroscopy alone or with supplementary transthoracic echocardiogram and TEE guidance.

## Intraprocedural Imaging Guidance

In performing transcatheter tricuspid valve intervention for TR, interventional imaging physician expertise in guidance of these complex procedures is of the utmost importance. Preprocedural CT is utilized for device selection and anatomical sizing. Intraprocedurally, 3-dimensional TEE remains the most utilized imaging modality by interventional imaging physicians, in addition to standard fluoroscopy. Clear and continuous communication between the interventional imaging physician and implanting physician is critical to procedural success. Live 3-dimensional multiplanar reconstruction imaging is used to optimize the trajectory of the delivery system and relationship between the TV leaflet and the device specific leaflet capture mechanisms. Common imaging protocols include utilization of MPR imaging and transgastric imaging (Figure 5). The transgastric short-axis view (∼30°) is used to aid positioning of the clip at target zone during TEER. This view helps to visualize all the TV leaflets, localize leaflet coaptation gap and regurgitation jet origin. Most importantly, this view enables continuous monitoring of the clip arm orientation to maintain perpendicularity to the line of coaptation.

**Figure 9:**
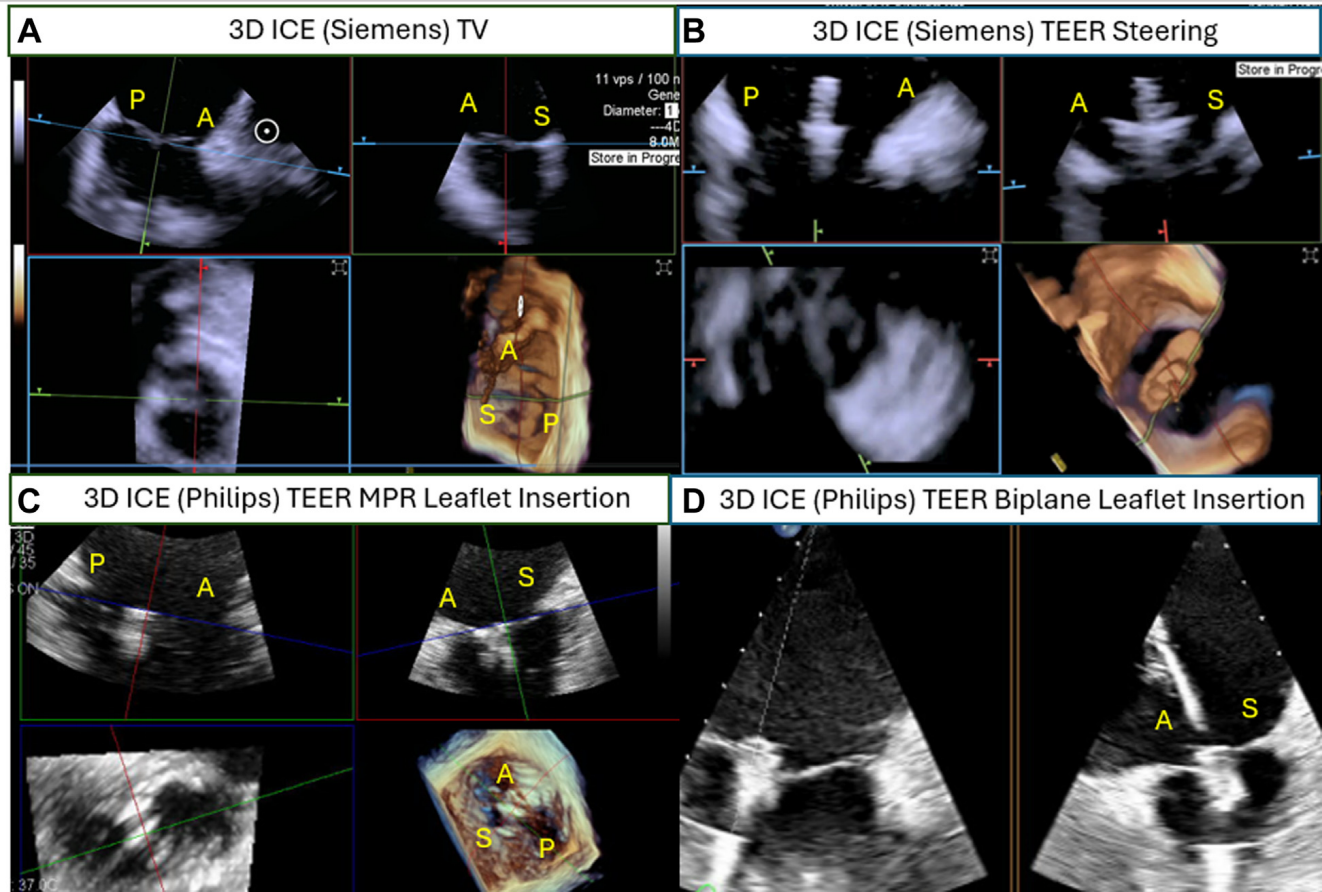
3D ICE to Guide Tricuspid TEER (A) 3-dimensional (3D) intracardiac echocardiography (ICE) (Siemens) showing the tricuspid valve (TV) anatomy. (B) 3D ICE is used to guide clip steering and clip arm orientation. (C) 3D ICE (Philips) multiplanar reconstruction (MPR) to assess leaflet insertion during Tricuspid-TEER. (D) 3D ICE biplane imaging demonstrating clip insertion on both the anterior (A) and septal (S) leaflets. P = posterior.

When TEE imaging is challenging, 3-dimensional intracardiac echocardiography may be used as a complimentary imaging tool.^81^, ^82^, ^83^ There are 3 different available 3-dimensional intracardiac echocardiography technologies ^84^ including AcuNav Volume (Siemens Healthiness), VeriSight Pro (Philips), and NuVision (Biosense Webster), and each of them has slightly different catheter diameter and field of view (Figure 9, Videos 4 and 5). The availability of 3-dimensional intracardiac echocardiogram can serve as a complimentary intraprocedural guidance to TEE in different transcatheter TV interventions, including TEER and transcatheter annuloplasty. However, 3-dimensional intracardiac echocardiogram is available in very limited sites in APAC (eg, Hong Kong) only, and the catheters are expensive (USD $4,500 to $5,500).

With the rapid growth in transcatheter tricuspid interventions, competent interventional echocardiographers are the key to the further expansion in this field. Therefore, dedicated training in interventional echocardiographers to guide these procedures is fundamental.^85^ However, there is currently no similar structured curriculum being developed within APAC,^86^ which could potentially limit the growth of tricuspid interventions in the region. Developing skills in interventional echocardiography will require not only having a structured curriculum to ensure competency, but also accessibility to novel imaging modalities and strong networking among the APAC structural imaging community.^86^

## Future Developments

### Clinical trial development

**Figure 10:**
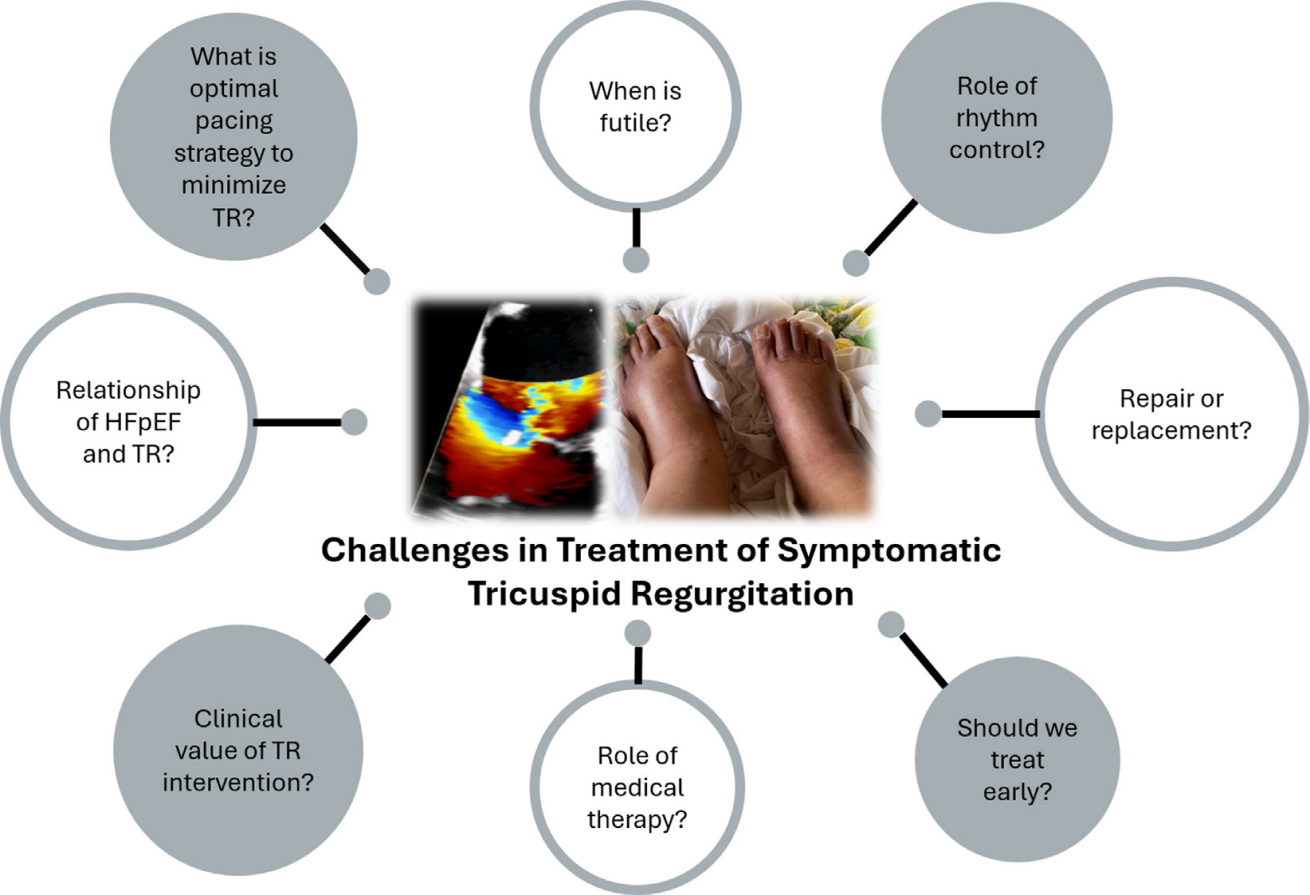
Future Challenges in Treatment of Symptomatic TR Future challenges in the treatment of tricuspid regurgitation (TR) include evaluating the role of atrial fibrillation rhythm control; the role of medical therapy; the relationship of TR with heart failure with preserved ejection fraction (HFpEF); the optimal pacing strategy to minimize TR; and the value, optimal timing, and optimal strategy of TR interventions.

Early tricuspid clinical trials such as TRILUMINATE failed to demonstrate a mortality benefit with invasive intervention on tricuspid regurgitation. However, TRILUMINATE demonstrated patients having improvement in Kansas City Cardiomyopathy Questionnaire scores and overall quality of life measures.^56^^,^^72^ As the field of transcatheter tricuspid clinical trials matures, further scientific evidence will be needed to refine patient selection, procedure type (repair vs replacement), as well as timing of intervention (eg, asymptomatic severe TR, symptomatic moderate TR) (Figure 10). The staging of right-sided heart failure is not yet clearly understood and may be used as a surrogate marker for future consideration of intervention for TR (Figure 10).

### Strategies to eliminate TR

There is a role for TEER and tricuspid valve replacement in the space of treating TR. Future TR therapeutic interventions may bear witness to more combined techniques in selected patients, eg, annuloplasty with TEER. In patients who have both mitral regurgitation and TR, timing of intervention, technique, and device selection remain patient specific.^87^^,^^88^

There remains uncharted territory in the field of electrophysiology and TR. CIED-related TR has garnered increasing clinical attention. Together with the rapid development in different pacing technique and leadless pacemaker, close collaboration with electrophysiologists to identify an optimal pacing strategy and to treat CIED-related TR patients is becoming more important.^89^ CIED lead extraction were performed mostly for lead malfunction or CIED-related infection, and rarely for treatment of CIED-related TR in APAC.^90^^,^^91^ Besides, lead extraction has been reported with mixed results in TR reduction.^89^ Due to the lack of evidence, there has been no unified approach and consensus statement in managing CIED-related TR in APAC. Earlier detection of CIED induce TR may allow for surgical or transcatheter guided lead positioning changes. Long-term, consideration of utilization of intraoperative imaging at the time of initial and subsequent right-sided lead/device placements may minimize patient’s risks of developing TR.

## Conclusions

TR is associated with significant morbidity and mortality. Remarkable advancements have been achieved in the past decade in transcatheter tricuspid valve intervention. There is heterogeneity of TR pathophysiology in the Asia Pacific region that requires a more targeted clinical approach based on the health care and socioeconomics of each region by workplace, community, and population health levels of implementation. As global clinical trials on tricuspid regurgitation continue the scientific path of discovery, the scientific community will gain a greater understanding of the disease mechanisms, the phenotypes of TR, and optimal timing for intervention and goal of preventing right heart failure.

## Funding Support and Author Disclosures

Dr So is a physician proctor for Abbott Structural Heart, Boston Scientific, Edwards and Medtronic. Dr Sung is a physician proctor for Abbott Structural Heart. Dr Meemook is a physician proctor for Abbott Structural Heart. Dr Wang is a consultant for Abbott, Edwards Lifesciences, and Materialise. Dr Tang has received speaker honoraria and served as a physician proctor, consultant, advisory board member, TAVR publications committee member, RESTORE study steering committee member, APOLLO trial screening committee member, and IMPACT MR steering committee member for Medtronic; has received speaker honoraria and served as a physician proctor, consultant, advisory board member and TRILUMINATE trial anatomic eligibility and publications committee member for Abbott Structural Heart; has served as an advisory board member for Boston Scientific and JenaValve; has served as a consultant and physician screening committee member for Shockwave Medical; has served as a consultant for NeoChord, Peija Medical, and Shenqi Medical Technology; and has received speaker honoraria from Siemens Healthineers. Dr Lee is a consultant for Abbott Structural Heart and HuiHe Medical. All other authors have reported that they have no relationships relevant to the contents of this paper to disclose.

## Appendix

Video 1 3-Dimensional Transesophageal Echocardiogram Multiplanar Reconstruction Showing the Implantation of the K-Clip Device Into Tricuspid Annulus

Video 2 Cine Movie Showing the Implantation of the Lux Valve in a Patient With Prior Mechanical Aortic Valve Replacement

Video 3 Right Atrial Angiogram Showing Absence of Contrast Reflux Into inferior Vena Cava After Implantation of a Heterotopic Valve at the inferior Vena Cava to Right Atrial Junction

Video 4 3-Dimensional intracardiac Echocardiogram (Siemens) Showing the Baseline Tricuspid Valve Morphology Before a Tricuspid Edge-to-Edge Repair Procedure

Video 5 3-Dimensional intracardiac Echocardiogram (Philips) Showing the Tricuspid Leaflet Grasping During Tricuspid Edge-to-Edge Repair Procedure
